# Enhanced corrosion resistance of zinc-containing nanowires-modified titanium surface under exposure to oxidizing microenvironment

**DOI:** 10.1186/s12951-019-0488-9

**Published:** 2019-04-16

**Authors:** Wen-qing Zhu, Shui-yi Shao, Li-na Xu, Wan-qing Chen, Xiao-yu Yu, Kai-ming Tang, Ze-hua Tang, Fa-ming Zhang, Jing Qiu

**Affiliations:** 1 Department of Oral Implantology, Affiliated Hospital of Stomatology, Nanjing Medical University, Nanjing, 210029 People’s Republic of China; 2 Jiangsu Key Laboratory of Oral Disease, Nanjing Medical University, Nanjing, People’s Republic of China; 3 Jiangsu Key Laboratory for Advanced Metallic Materials, Southeast University, Nanjing, People’s Republic of China

**Keywords:** Titanium, Corrosion behavior, Oxidizing condition, Macrophage

## Abstract

Titanium (Ti) and its alloys as bio-implants have excellent biocompatibilities and osteogenic properties after modification of chemical composition and topography via various methods. The corrosion resistance of these modified materials is of great importance for changing oral system, while few researches have reported this point. Recently, oxidative corrosion induced by cellular metabolites has been well concerned. In this study, we explored the corrosion behaviors of four common materials (commercially pure Ti, cp-Ti; Sandblasting and acid etching-modified Ti, Ti-SLA; nanowires-modified Ti, Ti-NW; and zinc-containing nanowires-modified Ti, Ti-NW-Zn) with excellent biocompatibilities and osteogenic capacities under the macrophages induced-oxidizing microenvironment. The results showed that the materials immersed into a high oxidizing environment were more vulnerable to corrode. Meanwhile, different surfaces also showed various corrosion susceptibilities under oxidizing condition. Samples embed with zinc element exhibited more excellent corrosion resistance compared with other three surfaces exposure to excessive H_2_O_2_. Besides, we found that zinc-decorated Ti surfaces inhibited the adhesion and proliferation of macrophages on its surface and induced the M2 states of macrophages to better healing and tissue reconstruction. Most importantly, zinc-decorated Ti surfaces markedly increased the expressions of antioxidant enzyme relative genes in macrophages. It improved the oxidation microenvironment around the materials and further protected their properties. In summary, our results demonstrated that Ti-NW-Zn surfaces not only provided excellent corrosion resistance properties, but also inhibited the adhesion of macrophages. These aspects were necessary for maintaining osseointegration capacity and enhancing the corrosion resistance of Ti in numerous medical applications, particularly in dentistry.

## Introduction

Titanium (Ti) and its alloys have been widely used in bone tissue engineering applications for decades because of their satisfactory biocompatibilities and excellent mechanical properties, especially for dental applications [1–3]. Among all of these properties, a rapid, strong and long-life bond between implant and bone is essential and fundamental [4]. A large number of studies have been dedicated to improving the osseointegration of endosseous implants. Many methods have been applied to modify the chemical composition and topography of Ti-implants, such as plasma spray, grit blasting, acid etching, anodization, etc. [5, 6]. All these modified titanium alloys are more effective in promoting osteoblast adhesion and function than conventional titanium implants, which could be utilized as novel potential materials in clinical applications.

Oral environment is a changing system and dental materials are prone to corrosion due to various factors, including body fluids (blood, plasma, amino acids and proteins), electrochemical activities of the implants (cathodic or anodic) and its interaction with bacteria, biological molecules and cells [7–9]. All these corrosive factors could alter the corrosion behavior and stability of titanium, increase corrosion susceptibility and accelerate the destruction of passivation film on titanium surface. It is known that higher corrosion rate indicates more ion release, which may interfere with cell metabolism in tissues around implants, and leads to implant failure [10, 11]. Recently, increasing evidences of inflammatory cell-induced corrosion of Ti dental implants have been reported [12, 13]. The mechanisms of this corrosion could be attributed to two reasons: On one hand, the inflammatory cells could attack invading bacteria and foreign bodies to resist external stimuli via acid and ROS [14]. On the other hand, inflammatory cells activated by implant are able to migrate and attach to its surface, release chemical species between cell membranes and metal, and then drive the corrosion [15]. Reactive oxygen species (ROS), specifically hydrogen peroxide (H_2_O_2_), is the major driver of the above process. The strong oxidizing effect of H_2_O_2_ creates local voltages between cell membranes and the alloy, which is sufficient to result in highly oxidizing conditions and raise the corrosion potential up into this region of voltage [16]. It has been reported that H_2_O_2_ in inflammatory cells could increase corrosion rates of CoCrMo and stainless steels alloys at relatively low concentrations in vitro [17], which is also the corrosion risk for Ti and its alloys. New technologies have been applied into the modification for dental implants in terms of the osseointegration, and many studies have investigated the corrosion behaviors of these modified materials. However, few studies have compared the corrosion resistance properties of these modified titanium materials, especially in a highly oxidizing condition.

In this study, commercially pure titanium (cp-Ti) was used as control surface. Sandblasting and acid etching (Ti-SLA), the most commonly used modification for clinical dental implants, with hierarchical micro-structured titanium surface, was used as experimental surface. Meanwhile, nanowires-modified titanium surface (Ti-NW) with bone-like nanowires structure on pure Ti surfaces, was also used as experimental surface.

Zinc coated materials have been widely used in biomedicine, and have been proved to improve osteoblastic differentiation [18, 19]. Zn ions could increase the stabilization of organic free radicals and accelerate the termination of free radical reactions via preventing the transfer of electrons to oxygen and organic molecules [20]. Thus, it is worthwhile to note the antioxidant property of zinc element and the corrosion behaviors of materials containing zinc element in oxidizing microenvironment. Here, acid-etched micro-structured titanium surface modified with zinc-containing nanowires was prepared as a new material based on the common Ti-NW surfaces (Ti-NW-Zn).

The novelty of this work was to compare the corrosion resistance of four modified dental implant materials (cp-Ti, Ti-SLA, Ti-NW and Ti-NW-Zn) with excellent osteogenesis and biocompatibilities under hydrogen peroxide environment created by macrophages. We aim to analyze the corrosion resistance characteristics of these most commonly modified titanium materials in a highly oxidizing condition and provide improved solutions for the oxidation corrosion of titanium materials.

## Materials and methods

### Sample and corrosion cell preparation

Pure titanium (99.5 wt% purity, Alfa Aesar, USA) disks, used as the control group, were polished at first. The SLA titanium surfaces, denoted as Ti-SLA samples, were obtained after being sandblasted and etched with solution containing HF/HNO_3_ (room temperature, 10 min) and solution containing H_2_SO_4_/HCl (80 °C in a water bath, 30 min). Nanowire modified titanium surfaces, denoted as Ti-NW samples, based on cp-Ti, were etched firstly with 0.4% hydrofluoric acid for 30 min and washed with distilled water. Subsequently, these samples were soaked in a 10% NaOH aqueous solution (70 °C in a water bath, 15 min). Hydrothermal products were rinsed with distilled water and air dried. Half of the Ti-NW samples were then soaked in a 10 mM ZnSO_4_ aqueous solution (70 °C in a water bath, 15 min). After being washed and dried, the obtained samples were denoted as Ti-NW-Zn samples.

### Characterization of the corrosion surface

Morphology, roughness and elemental composition of different alloy surfaces co-cultured with or without macrophages were observed using scanning electron microscopy (SEM; Sirion 200, Philips, Eindhoven, Netherlands) and 3D surface topography instrument (Nanjing RanRui Technology Co., Ltd, China). The different alloys were removed from the medium after 5-day culture either with macrophages or not. They were lightly rinsed in sterile deionized water, washed twice in PBS, underwent ultrasonic bath with distilled water for 5 min and then fixed in 2% glutaraldehyde for 30 min. Afterward, the samples were washed three times in PBS and dehydrated via a series of graded ethanol solutions (50, 70, 80, 90, and 100%). Finally, the coupons were analyzed by SEM at 15 kV and 3D surface topography instrument.

### Tribology tests of different surfaces

The ball-disk contact mode reciprocating friction and wear test were carried out using a linear reciprocating tribometer to investigate the tribological properties of different alloy surfaces in a dry environment. These friction and wear tests were performed in ambient air at room temperature under the normal force of 30 N, a set frequency of 3 Hz and a friction displacement of 10 mm. The test time was 20 min to ensure stable wear of the sample material. The size of all four materials was 20 mm × 20 mm × 3 mm, and Tungsten Carbide (WC) was used as the grinding ball with the size of φ 6.35 mm. The test materials and the grinding balls were cleaned by ultrasonic cleaning with ethanol and dried for use. The three-dimensional appearances of wear scar on the four surfaces were observed by 3D surface topography instrument (Nanjing RanRui Technology Co., Ltd, China). The coefficient of friction (COF) data were obtained by Multi-Function Tribometer (MFT-3000, Rtec Instruments, CA, USA) and analyzed by software RtecViewer (Nanjing RanRui Technology Co., Ltd, China). The experiment was performed in triplicate.

### Surface analysis

X-ray photoelectron spectroscopy (XPS) (PHI 5000 VersaProbe, Ulvac-Phi, Japan) was used to determine the elemental components and chemical states present on the surfaces of three alloy specimens before and after the pretreatment utilizing a monochromatic Al Kα electrode (15 kV, 150 W and 45° take-off angle). High-resolution spectra were obtained using pass energies of 160 and 40 eV, respectively. Reference binding energies of each element were obtained from the National Institute of Standards and Technology XPS Online Database (http://srdata.nist.gov/xps/). All spectral features were referenced to the binding energy of adventitious carbon (284.8 eV). Quantitative analysis of the surface chemical composition was performed using peak areas and atomic sensitivity factors.

### Electrochemical corrosion test

Before testing, the metal specimens were carefully mounted in self-cured epoxy resin, exposing their surfaces, and ultrasonically cleaned in ethanol and de-ionized water. Corrosion tests were performed using an electrochemical potentiostat (CS310H, Wuhan Corrtest Instrument Co., Ltd, China) via a test cell with the mounted specimen as the working electrode, a high-purity platinum wire as the counter electrode, and Ag/AgCl as the reference electrode. Corrosion tests were performed in triplicate for each alloy, before and after the pretreatment, in phosphate buffered saline, at 37 ± 0.5 °C. Each specimen was allowed to reach a steady open circuit potential (E_corr_) for 2 h, after which a 10 mV amplitude sine wave potential was applied through a frequency range of 1000 kHz to 10 mHz. Electrochemical impedance spectroscopy (EIS) tests were implemented using the dedicated PowerSine software. The acquired data, including Nyquist plot, Bode |Z|, and Bode Phase diagrams, were analyzed and fitted using an appropriate equivalent circuit by the ZsimpWin software. The E_corr_ was recorded and then a potentiodynamic polarization test was initiated within a scanning range from 400 to +1600 mV (versus reference electrode) at a sweep rate of 1 mV/s. The acquired polarization curves were analyzed using the curvetting routine of the dedicated PowerSuite soware (CS Studio 5, Wuhan Corrtest Instrument Co., Ltd, China) to calculate the corrosion current (I_corr_) and the polarization resistance (R_p_) of the materials.

### Determination of H_2_O_2_ content

Culture supernatants of each well with different treatments were collected. The content of H_2_O_2_ was analyzed with the Hydrogen Peroxide assay kit (Beyotime, S0038) according to the manufacturer’s protocols. In brief, test tubes containing 50 µl of test solutions were placed at room temperature for 30 min and measured immediately with a spectrophotometer at a wavelength of 560 nm. Absorbance values were calibrated to a standard curve generated with known concentrations of H_2_O_2_.

### Cell culture

Osteoblast-like cell line MC3T3-E1 were purchased from the Chinese Academy of Sciences Cell Bank (Shanghai, China). The cells were cultured in α-Minimum Essential Medium (α-MEM; Gibco, USA) containing 10% fetal bovine serum (FBS; Gibco, USA) and 1% penicillin/streptomycin (Gibco, Life Technologies, Carlsbad, CA, USA) in a humidified atmosphere of 5% CO_2_ and 95% air at 37 °C. Murine macrophage-like RAW264.7 cells, obtained from the Cell Bank of the Chinese Academy of Sciences (Shanghai, China), were cultured in Dulbecco’s Modified Eagle’s Medium (DMEM, Sigma-Aldrich LLC, St. Louis, MO) containing 10% fetal bovine serum (FBS; Gibco, USA) and 1% penicillin/streptomycin (Gibco, Life Technologies, Carlsbad, CA, USA) in a humidified atmosphere of 5% CO_2_ and 95% air at 37 °C. The medium was changed every 2 days. Cell passage was conducted at a ratio of 1:4 every 3–4 days until 80% of confluence.

### Cell proliferation assay

The four materials (cp-Ti, Ti-SLA, Ti-NW and Ti-NW-Zn) samples were prepared and applied into 96-well plates. Subsequently, MC3T3-E1 cells and RAW264.7 cells were seeded (2 × 10^3^ cells/well) onto each surface of alloy and maintained in a 5% CO_2_ incubator at 37 °C for 1, 3 and 6 days, respectively. 100 μl of fresh medium containing 10 μl of CCK-8 solution (Beyotime, Shanghai, China) were added in each well, followed by incubation at 37 °C for another 2 h. Thereafter, the absorbance at the wavelength of 450 nm of each well was recorded with a microplate spectrophotometer (Spectramax 190, CA, USA). Cell proliferation was determined by the absorbance in each well.

### Cell adhesion and spreading assay

For cell adhesion and spreading assay, RAW264.7 cells (1 × 10^4^ cells per well) were seeded on one specimen of each alloy before and after the pretreatment. After cell culture for 4 h, each sample was rinsed with PBS, and then fixed with 4% paraformaldehyde in PBS at room temperature for 10 min. Afterwards, each sample was stained with Rhodamine phalloidin (Cytoskeleton, USA) at room temperature in dark for 30 min and then 4′,6′ -diamidino-2-phenylindole (DAPI) (Beyotime, Shanghai, China) for 30 s. The cell spreading morphology was observed in three randomly selected fields per sample under a laser scanning confocal microscope (LSM710, Zeiss, GER) at 100× and 200× magnification.

### Western blotting

The four materials (cp-Ti, Ti-SLA, Ti-NW and Ti-NW-Zn) samples were prepared and applied into 6-well plates. Subsequently, MC3T3-E1 cells (2 × 10^5^ cells/well) were seeded onto each surface of alloy and cultured for 7 days. Protein samples were extracted from cells by RIPA buffer after cell washing with pre-cooled PBS. After the electrophoresis, protein samples were transferred to a polyvinylidene fluoride membranes (PVDF; Millipore, Billerica, MA, USA). Membranes were blocked for 1 h at room temperature (5% nonfat dry milk, in Tris-buffered saline containing 0.1% Tween-20; TBST). They were subjected to incubation with primary antibodies against Runx2 (12556; CST, Beverly, MA, USA), OSX (ab22552; Abcam, Cambridge, MA, USA) or GAPDH (BM0627, Boster, China) overnight at 4 °C. After TBST wash for three times, membranes were incubated with secondary antibodies (ZB-2301; Goat anti-Rabbit IgG, ZSGB-BIO, China) for 2 h, followed by chemiluminescence exposure using ECL Western Blot Kit (Millipore, USA). The background was subtracted, and the signal of each target band was normalized to that of the GAPDH band. Protein level was quantified using the Image J software k1.45 (version k1.45; National Institutes of Health, Bethesda, MA, USA). The experiment was performed in triplicate.

### Flow cytometry

The four materials (cp-Ti, Ti-SLA, Ti-NW and Ti-NW-Zn) samples were prepared and applied into 24-well plates and then RAW264.7 cells were seeded onto different topographies (1.5 × 10^5^ cells/well). Macrophage surface markers CD11c (M1) and CD206 (M2) were detected by flow cytometry for evaluating the different phenotypes. After 3-day incubation, cells were scraped off, centrifuged, and resuspended in purified anti-mouse CD16/32 (Biolegend, San Diego, CA, USA) for 10 min at 4 °C to block the nonspecific antigens. Then, RAW264.7 cells were incubated with PE-conjugated CD11c (Biolegend) and PerCP-conjugated CD206 (Biolegend) for 30 min at 4 °C. After washing twice in PBS, RAW264.7 cells were resuspended in PBS and analyzed on a flow cytometer (NovoCyte; ACEA Biosciences, San Diego, CA, USA). All samples were analyzed in triplicate.

### Enzyme-linked immunosorbent assays (ELISA)

Un-activated RAW 264.7 cells were plated onto different topographies in 6-well plates and cultured for 3 days. Then the supernatants were harvested from each well. To observe the pro-inflammatory and anti-inflammatory cytokine release, levels of M1 macrophage markers (IL-6; FMS-ELM006, CST, China) and M2 macrophage markers (IL-10; FMS-ELM009, CST, China) in the supernatants were measured by enzyme-linked immunosorbent assays (ELISA, CST, China) for cytokine profiling following the manufacturer’s protocol. All samples were analyzed in triplicate.

### RNA isolation and quantitative reverse transcription

Polymerase chain reaction (PCR) was used to examine the genes expression of antioxidant enzyme system in macrophages on different alloy surfaces. Cells (2 × 10^5^ cells per well) were seeded in a 6-well plate and cultured for another 5 days with complete culture solution. According to the manufacturer’s protocol, the total RNA was extracted with TRIzol reagent (Invitrogen, Carlsbad, CA, USA) and then reversely transcribed (Takara, Tokyo, Japan). Quantitative real-time PCR (qPCR) was performed with SYBR^®^ Premix Ex TaqTM II (RR820A; Takara) in a total reaction volume of 20 μl containing 2.0 μl of cDNA template per well by the 7300 Real-Time PCR Detection System (Applied Biosystems, Foster City, CA, USA). β-Actin (B661302-0001; Sangon, China) was selected as an internal reference for normalization. The primer sequences were shown in Table 1. When the amplification period ended, melting curve analysis was performed to confirm the specificity of the amplicon. Relative quantification of gene expression was calculated using 2^−ΔΔCt^ equation. All data derived using qPCR was conducted in least three independent biological samples.

**Table 1:** Primer sequences of target genes for real-time PCR in this study

Gene	Primer sequence (F: forward; R: reverse)	Product size (bp)
Catalase (CAT)	F: GCAGATACCTGTGAACTGTCCCT	172
	R: TTACAGGTTAGCTTTTCCCTTCG	
β-Actin	F: GTGCTATGTTGCTCTAGACTTCG	174
	R: ATGCCACAGGATTCCATACC	

### Statistical analysis

Data were analyzed by SPSS 22.0 software (SPSS, Inc., Chicago, IL, USA). Differences among multiple groups were analyzed by the standard analysis of variance (ANOVA), followed by the Bonferroni’s post hoc test. The Student’s independent-samples *t* test was used to compare differences in numerical results before and after the pretreatment. The probability level for statistical significance was set at α = 0.05.

## Results and discussion

### Microstructural observation

**Fig. 1:**
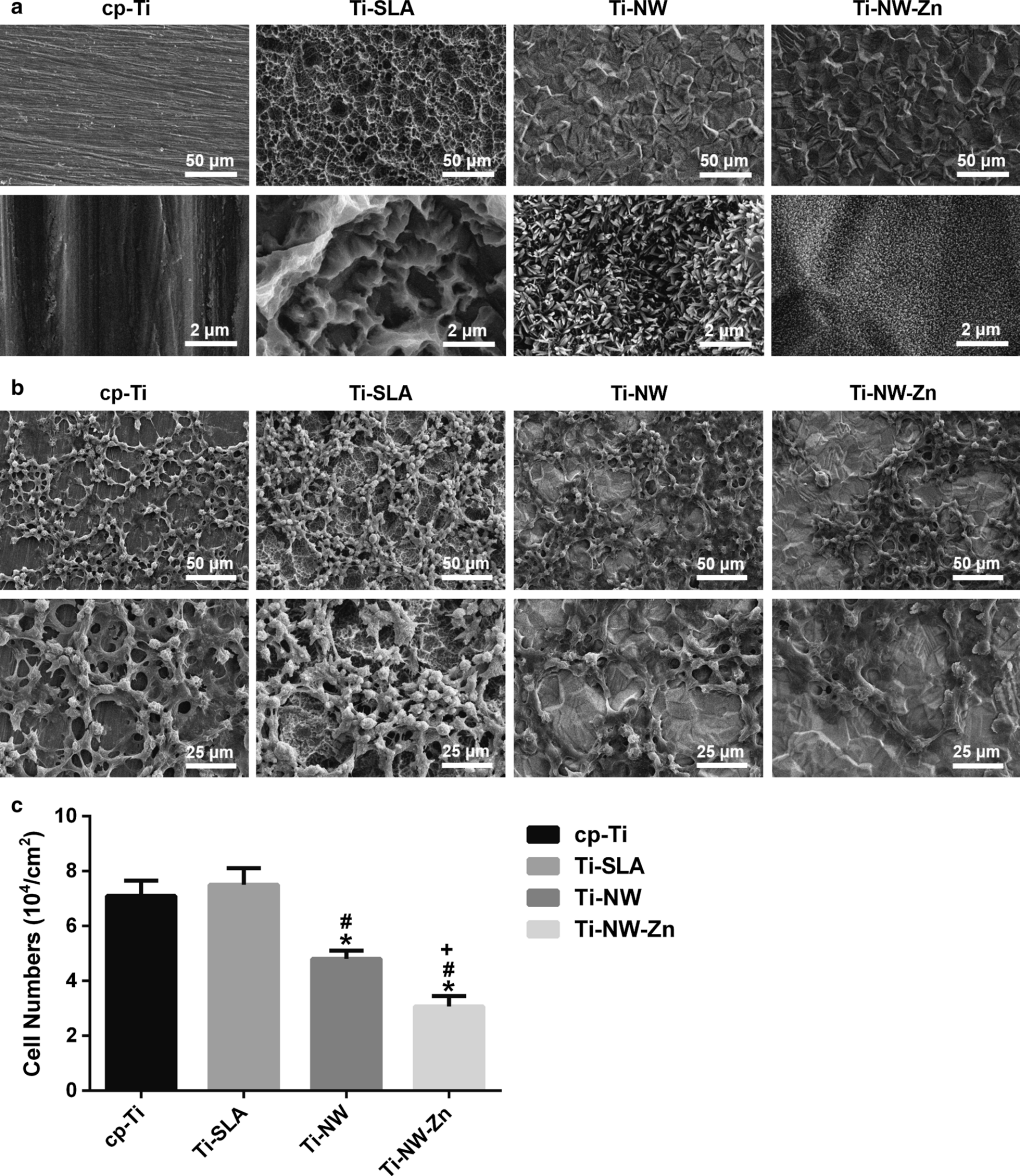
SEM images of the samples before and after co-culture with macrophages: **a** cp-Ti, Ti-SLA, Ti-NW and Ti-NW-Zn surfaces without cell attachment (magnification ×500 and ×10,000); **b** cp-Ti, Ti-SLA, Ti-NW and Ti-NW-Zn surfaces with cell attachment (magnification ×500 and ×10,000); **c** Quantitative analysis for the number of macrophages on each surface. Results are presented as Mean ± SD, * *p *< 0.05 when compared with the cp-Ti group; ^#^*p *< 0.05 when compared with the Ti-SLA group; ^+^*p *< 0.05 when compared with the Ti-NW group

Results showed the surface views of cp-Ti, Ti-SLA, Ti-NW and Ti-NW-Zn samples before and after co-cultured with macrophages (Fig. 1). All substrate surfaces, except for the smooth cp-Ti, exhibited microscale rough structures under lower magnification (Fig. 1a). However, Ti-SLA surface displayed rougher topography than Ti-NW and Ti-NW-Zn surfaces because of its unique hierarchical microstructures. Under higher magnification, micro-pits and sharp edges could be observed on the Ti-SLA surface. The hydrothermal method has been proved to be a facile and cost-effective approach to obtain nanostructured titanate layers on Ti surfaces under relatively high pressure and temperature [21]. The composition of nanostructured titanate layers on Ti surfaces is H_2_Ti_2_O_5_∙H_2_O and Na_2_Ti_2_O_5_∙H_2_O after hydrothermal method [22]. By changing the concentration of NaOH to 10 mM according to previous studies [23], the surfaces of Ti-NW and Ti-NW-Zn samples in our study were covered with nanowire-like structures. However, no visible changes could be found on neither the Ti-NW nor the Ti-NW-Zn surfaces. The similar nanostructures were also shown in the SEM images, indicating that the secondary ZnSO_4_ hydrothermal treatment could not change the microstructures. As shown in Fig. 1b, the macrophages on each surface represented as a spherical structure. Besides, the number of cells on the surfaces of Ti-NW and Ti-NW-Zn were significantly less than that on cp-Ti and Ti-SLA, while no obvious difference was observed between cp-Ti and Ti-SLA. Furthermore, cells on the surfaces of Ti-NW-Zn were the least among all the groups (Fig. 1c). These results suggested that different surface structures could alter material biocompatibility on cell behaviors and might influence cells fates.

### Tribological performance of four different surfaces

**Fig. 2:**
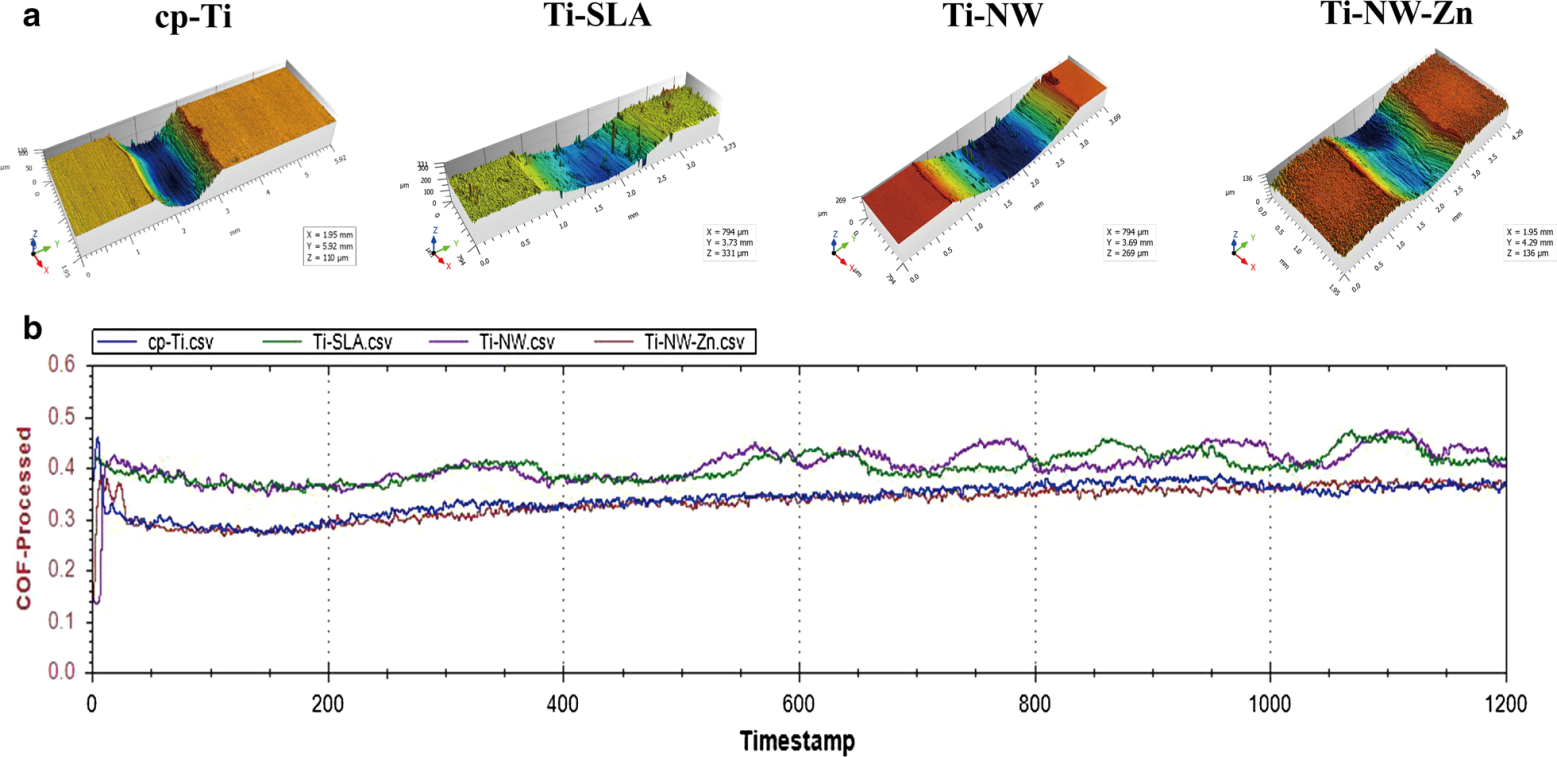
Tribological performance of four different surfaces: **a** three-dimensional appearances of wear scar of the four surfaces; **b** the coefficient of friction (COF) of the four surfaces

The three-dimensional appearances of wear scar on the four surfaces were observed to investigate the effects of fretting wear on different surface damages. The models in Fig. 2a showed that the depth and width of the wear scar on Ti-SLA and Ti-NW surfaces were deeper and wider than that of cp-Ti and Ti-NW-Zn surfaces, indicating that Ti-SLA and Ti-NW surfaces were more vulnerable to wear.

Furthermore, Dynamic friction spectra as a function of time elapsed (in seconds) for four separate surfaces sliding tests were explored. The coefficient of friction (COF) of all four surfaces started with an unstable value because of initial acceleration, and gradually came to a steady state value (Fig. 2b). This run-in mechanism has been observed in other studies and recognized as a polishing mechanism between two contact surfaces that have different surface roughness [24]. It is believed that, after the wear has progressed and the track has become more polished, the COF data will become stabilized [25].

Our study illustrated that COF for Ti-SLA and Ti-NW surfaces were higher than that of cp-Ti and Ti-NW-Zn surfaces, while COF between Ti-SLA and Ti-NW surfaces and COF between cp-Ti and Ti-NW-Zn surfaces had no difference. No oscillations could be found for all four spectra after COF stabilization in the early stage. It is indicated that no instrumental failure occurred and no surface cracking or spalling was present during this stage. However, the COF value for Ti-SLA and Ti-NW surfaces swayed with the prolongation, indicating a trend of change of the surface structure, whereas the COF for cp-Ti and Ti-NW-Zn surfaces were still stable. It is widely believed that film roughness exerts an important role in the COF. Generally speaking, a rougher film results in larger contact area and more asperity collisions [26]. Given that all surfaces were tested against Tungsten Carbide at 30 N load in dry air, 3 Hz frequency and 10 mm displacement. The high COF for Ti-SLA and Ti-NW surfaces could be explained by the interaction mechanism between two contact surface alterations because of their high surface roughness. Roughness of surface further contributed to low lubrication of surfaces and made the oxide more vulnerable to wear. Also, the existence of zinc chemical bonds changed the surface frictional energy dissipation and the coefficient of friction, which was different from the Ti-NW surface and close to the smooth cp-Ti surface. The deep mechanisms of tribological performance are needed to be further studied.

### Oxidation environment and roughness change

**Fig. 3:**
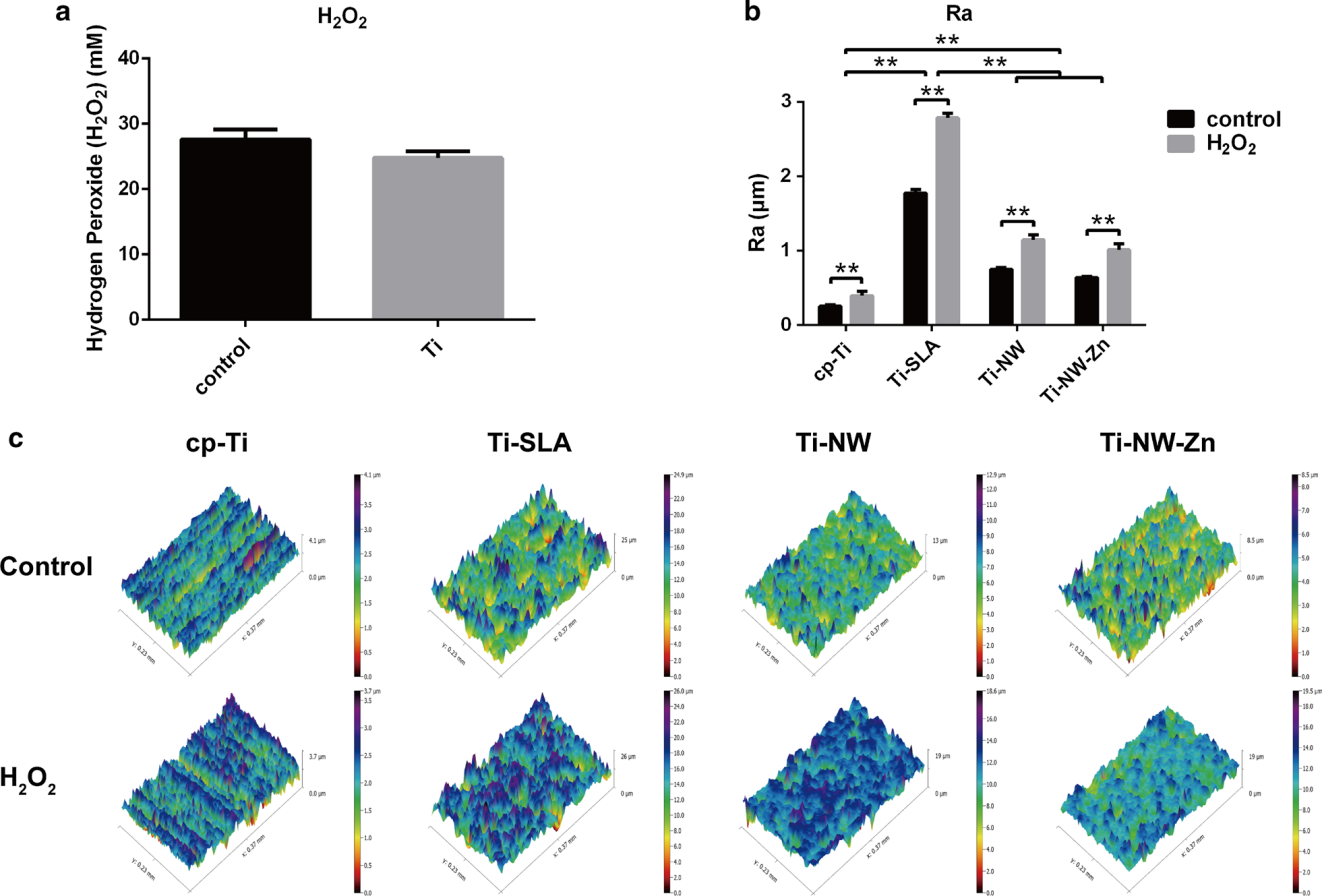
Oxidation environment and roughness change: **a** determination of H_2_O_2_ content from macrophages cultured on titanium surfaces or not; **b** roughness for sample surfaces before and after cultured with macrophages; **c** 3D surface topography images for sample surfaces before and after cultured with macrophages

To explore the oxidation environment around titanium surfaces adhered by macrophages, we determined the H_2_O_2_ content. Figure 3a showed the release of H_2_O_2_ from macrophages following the 5-day culture. The result showed that H_2_O_2_ content of each group reached up to 30 mM and there was no difference between the control group and titanium surface group. Highly oxidizing condition was proved to raise the open circuit potential (OCP) and further alter the local voltages and currents between cell membranes and the alloy [27]. It is sufficient to change the surface structures of oxide layer on metal substrates, and might further influence the stability of the passivation film formed on titanium surface.

For a better understanding of the highly oxidizing condition effects on different surfaces in our present study, we further detected the 3D surface topography images and roughness of the four surfaces before and after macrophage co-culture (Fig. 3b, c). After removing cells seeded on samples, a rougher structure and morphological features were revealed than those without cells attachment. It has been reported that rougher surfaces might induce more cell adhesion [28]. Our results suggested that macrophages attachment might change the surface structures of oxide layer on titanium substrates via creating a highly oxidizing condition and made the surface rougher to induce more cells adhesion.

### Surface analysis

**Fig. 4:**
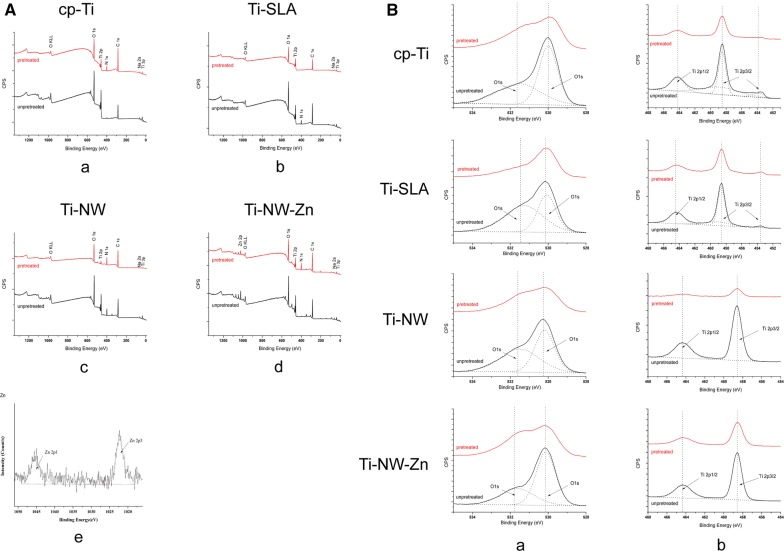
XPS analysis of the four different surfaces with or without macrophage attachment. **A** XPS wide scan spectra analysis: **a** cp-Ti; **b** Ti-SLA; **c** Ti-NW; **d** Ti-NW-Zn. **e** XPS high-resolution spectra of the Zn element on Ti-NW-Zn surface; **B** XPS high-resolution spectra analysis: **a** O 1s peaks for four different surfaces before and after attached to macrophages; **b** Ti 2p peaks for four different surfaces before and after attached to macrophages

To better understand the changes in the surface structures of oxide layer on titanium substrates in highly oxidizing condition, X-ray photoelectron spectroscopy (XPS) was conducted to detect the surface elemental components and chemical states. Figure 4A(a–d) showed the XPS wide scan spectra analysis of the four different surfaces with or without macrophage attachment. Titanium (Ti) and oxygen (O) elements were shown to be present on cp-Ti, Ti-SLA, Ti-NW and Ti-NW-Zn surfaces, while Zinc (Zn) element was only present on surfaces of Ti-NW-Zn samples. The Sodium (Na) element was most likely originated from the NaOH hydrothermal reaction, while adventitious Carbon (C) peaks were probably attributed to contamination. Figure 4A(e) showed the XPS high-resolution spectra of the Zn element on Ti-NW-Zn surface. The energies of the Zn 2p3 peak at 1021.7 eV and 1044.8 eV could be assigned to Zn 2p1 and did not shift with the depth under investigation. The binding energy of the Zn 2p3/2 peak was located at 1021.7 eV, a perfect fit with Zn^2+^ in ZnO. All above results indicated that Zn element was indeed embedded into the titanium substrate. Many studies have found the Zn element is essential for a variety of biological systems, including bone metabolism and dental development [29, 30]. Recent research has also considered it as an antioxidant to defend organism away from reactive oxygens [31]. Thus, the presence of Zn on the titanium substrate might be beneficial to osteogenic properties and contributes to improve the oxidizing environment. Furthermore, both O 1s and Ti 2p peak intensities markedly decreased in all the four different surfaces pretreated with macrophages compared to those without macrophages attachments. Also, the peak intensities on Ti-NW-Zn surface was the weakest.

From XPS high-resolution spectra analysis in Fig. 4B, the O 1s peaks for four different surfaces before and after attachment to macrophages were all attributed to two peaks at 531.5 eV (OH−) and 530 eV (O2−) (Fig. 4B(a)). Ti 2p peaks were all attributed to two peaks at 464.6 eV (TiO_2_) and 458.5 eV (TiO_2_) (Fig. 4B(b)). The results revealed a remarkable decrease in both the oxygen and titanium dioxide contents on all the four surfaces after co-culture with macrophages, and they showed the lowest levels on the Ti-NW-Zn surfaces.

Recently, studies have presented new evidences of direct inflammatory cell-induced (ICI) corrosion of metal alloy implant surface [13]. Researchers hypothesized that different classes of cells from both the skeletal and immune systems (such as phagocytic cells, osteoclasts, macrophages and foreign giant cells) may directly attack surfaces of metal implants, creating corrosion-like pattern that is usually found on the non-contacting regions of retrieved components [17]. Some in vivo and in vitro studies also supported that monocytes can differentiate into osteoclasts and corrode metallic surfaces made of titanium and stainless steel [32]. Macrophages, as one of the inflammatory cells, has also been reported to significantly increase corrosion susceptibility of implants by secreting reactive oxygen species (ROS), specifically hydrogen peroxide (H_2_O_2_).

In this article, unobvious reduction of O 1s and Ti 2p peak intensities in Ti-NW-Zn indicated that the surface structure of oxide layer on Ti-NW-Zn did not markedly change in highly oxidizing condition compared with other three surfaces. It might be attributed to zinc element. Nevertheless, reactive fresh zinc surface under exposure to the atmosphere subsequently formed dense, adherent corrosion byproducts, leading to 10- to 100-fold decrease in the rate of corrosion than ferrous materials depending on the environment. These corrosion products develop rapidly on metal surface as the coating and act as an additional barrier between the metal and the environment, which might make it a well-suited corrosion protective coating for metal products [33]. Thus, in highly oxidizing condition, zinc might limit reactions occurring on the titanium oxide layer of Ti-NW-Zn, which protects the titanium substrates. To confirm the potential excellent corrosion resistance of Ti-NW-Zn surface, we further detected the corrosion behavior of Ti-NW-Zn material compared with other surfaces under hydrogen peroxide environment to explore its sensitivity to H_2_O_2_ induced corrosion attack.

### Corrosion behavior

To simulate a similar oxidizing condition and better understand the effects of H_2_O_2_ on the corrosion behavior of the four materials, samples were immersed in phosphate buffered saline (pH 7.4 at room temperature) between 0 mM and 30 mM H_2_O_2_ (according to the H_2_O_2_ content determined above). Electrochemical Impedance Spectroscopies (EIS) was evaluated. During the last decade, EIS has been proven to be advantageous for the characterization of various oxide films on metal surfaces. This technique requires non-destructive procedures in the open circuit mode, i.e., neither oxidation nor reduction is forced to take place.

**Fig. 5:**
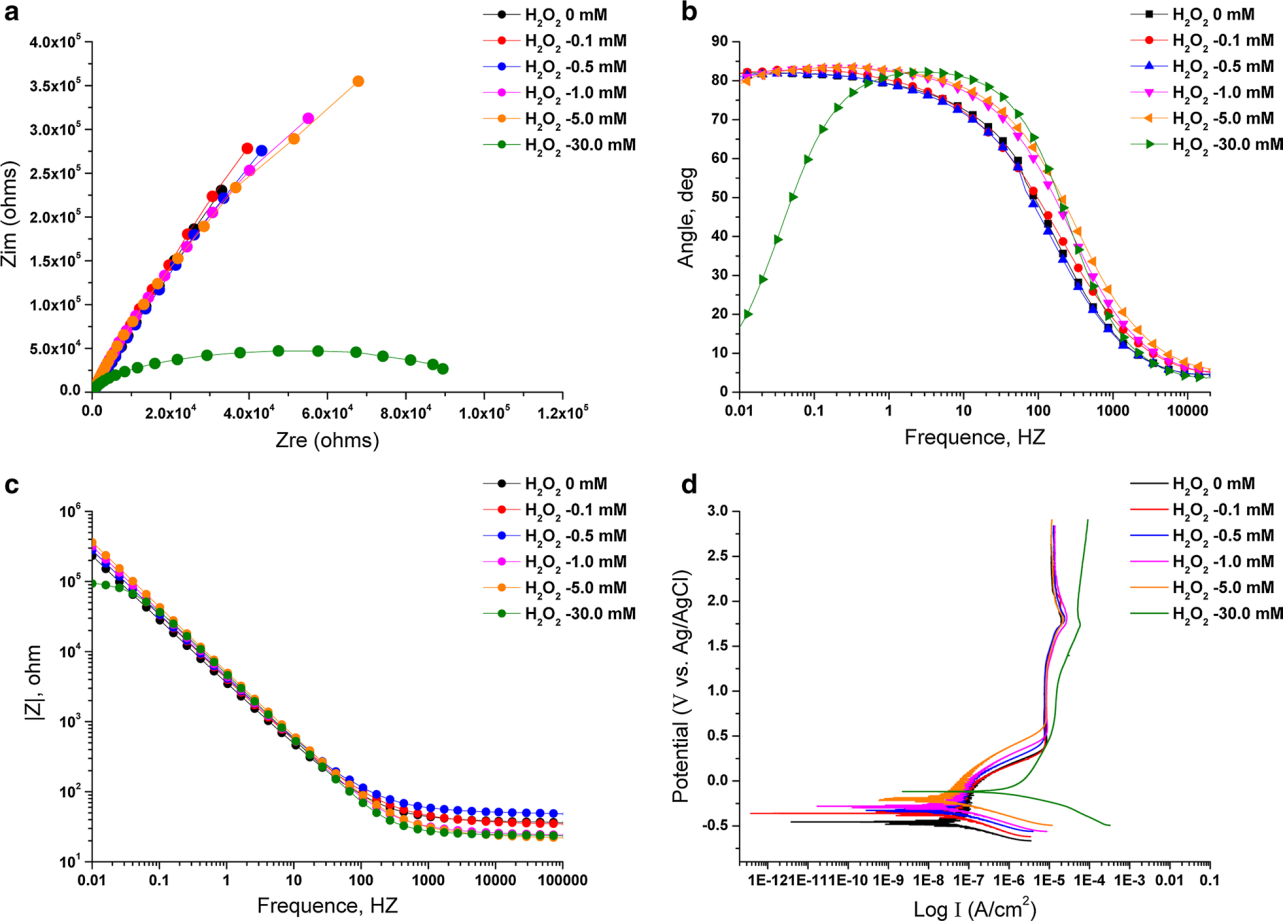
**a** The Nyquist plot diagram for cpTi coupons immersed in solutions with additions of H_2_O_2_ between 0 and 30 mM; **b**, **c** Typical Bode Phase and Bode |Z| diagrams for cp-Ti immersed in solutions with additions of H_2_O_2_ between 0 and 30 mM: **b** Bode phase angle diagrams and **c** Bode |Z| diagrams; **d** typical diagrams of the potentiodynamic polarization for cp-Ti surfaces immersed in solutions with additions of H_2_O_2_ between 0 and 30 mM

Representative EIS data were showed in Fig. 5a, and all of the specimens yielded data tracing a single semicircle. The diameters of the impedance loops markedly decreased in a dose-independent manner when the cp-Ti coupons were immersed in solutions added H_2_O_2_. Especially, samples treated with 30 mM H_2_O_2_ showed the lowest impedance diameters compared to the other samples. These results implied an increase in the chemical reactivity and corrosion rates of the cp-Ti specimens as a consequence of exposure to H_2_O_2_. The more H_2_O_2_ released, the weaker resistance to corrosion presented.

**Fig. 6:**
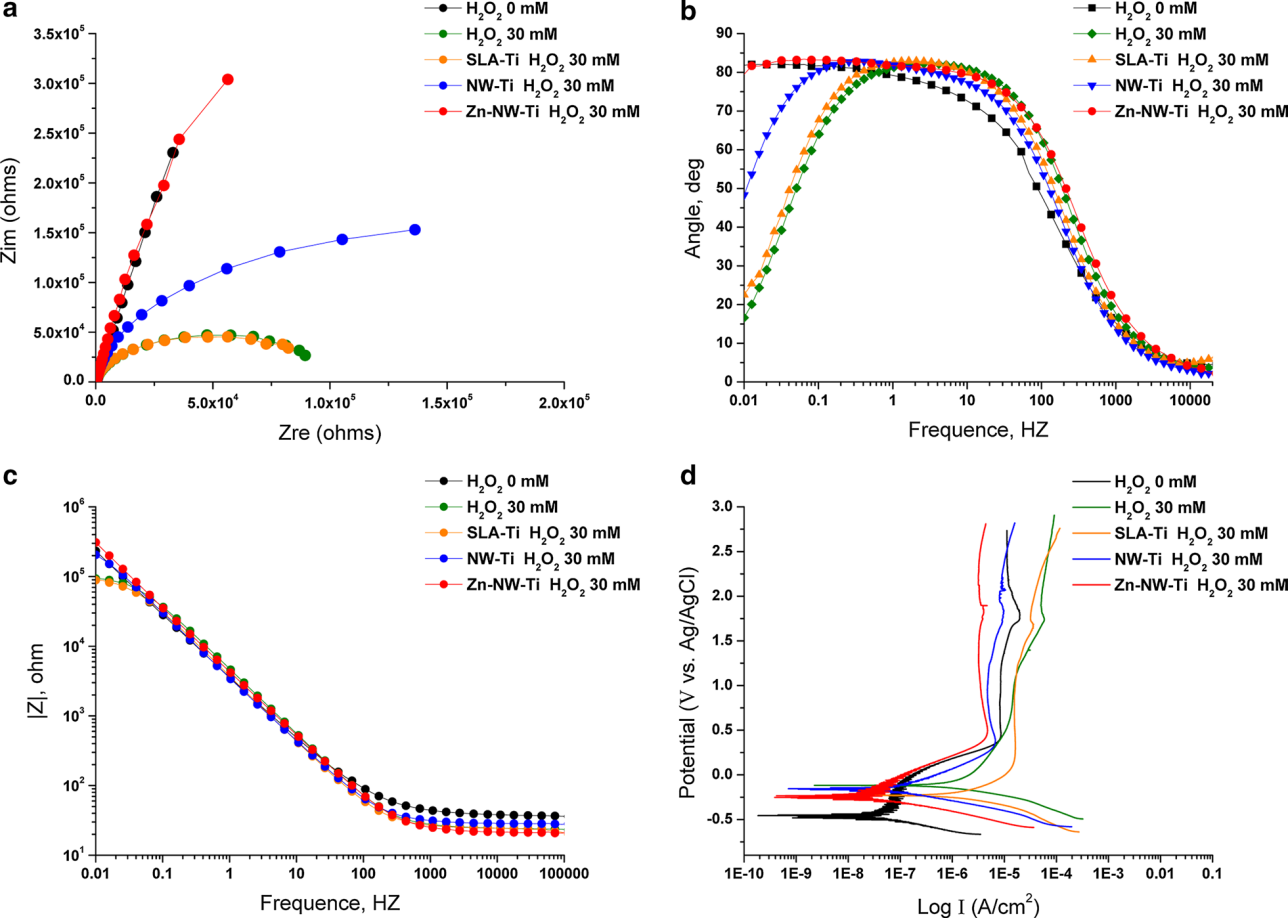
**a** The Nyquist plot diagram for different materials (cp-Ti, Ti-SLA, Ti-NW and Ti-NW-Zn) exposed to the corrosion solutions with 30 mM H_2_O_2_; **b**, **c** Typical Bode Phase and Bode |Z| diagrams for different materials immersed in solutions with 30 mM H_2_O_2_: **b** Bode phase angle diagrams and **c** Bode |Z| diagrams; **d** Typical diagrams of the potentiodynamic polarization for different materials immersed in solutions with 30 mM H_2_O_2_

We further explored the impedance diameters for different materials exposed to the corrosion solutions with H_2_O_2_. As shown in Fig. 6a, all specimens approximately revealed only one semicircle. The diameter for cp-Ti, Ti-SLA and Ti-NW samples immersed in 30 mM H_2_O_2_ were significantly reduced than the control group, while the diameter of the semicircle for Ti-NW-Zn sample had no difference with the PBS group. The EIS data revealed a concomitant weakening of corrosion resistance when coupons exposure to H_2_O_2_ environment. All of these results illustrated that Ti-NW-Zn surface showed a relatively inactive corrosion behavior under exposure to H_2_O_2_ compared with other surfaces in this study.

Representative electrochemical impedance data in the form of Bode Phase and Bode |Z| diagrams were showed in Fig. 5b, c. The results showed a large drop in low-frequency impedance (0.01 Hz, associated with the oxide resistance, based on a Randle’s circuit approximation of the interface) and narrowing of the frequency range of the phase angle with a low dose of H_2_O_2_. The decrease in impedance modulus at the lowest frequency as a function of exposure concentration indicated the corrosive effect of H_2_O_2_ on cp-Ti surfaces. 30 mM H_2_O_2_ incubation significantly decreased the low-frequency impedance of the surfaces compared with other groups. A higher phase shift at a lower frequency in Bode phase plots is indicative of a good passive film [34]. Therefore, compared with samples immersed in H_2_O_2_ free solutions, passive film formed on the cp-Ti became defective or unstable after immersion in H_2_O_2_ solutions. These results indicated that H_2_O_2_ did increase the corrosion susceptibility of titanium materials.

Focused on the corrosion resistance of different materials exposure to H_2_O_2_, our present research clearly demonstrated the properties of oxide films on the four different surfaces. The electrochemical impedance data in the form of Bode Phase and Bode |Z| diagrams were displayed in Fig. 6b, c. The cp-Ti immersed in PBS alone, as a control group, showed phase angles close to 83° at 0.01 Hz. After immersion in solutions with 30 mM H_2_O_2_, the phase angle of cp-Ti, Ti-SLA and Ti-NW samples at the lowest frequency of 0.01 Hz exhibited different degrees of decline (the drop of phase angles was cp-Ti > Ti-SLA > Ti-NW). Surprisingly, phase angles of Ti-NW-Zn had almost no change and was much higher than the other groups when exposed to 30 mM H_2_O_2_. Similarly, the total impedance magnitude at the lowest frequency for cp-Ti, Ti-SLA and Ti-NW samples immersed in solutions with 30 mM H_2_O_2_ showed a significant drop compared with the cp-Ti immersed in PBS. Identically, H_2_O_2_ exposure had little effect on Ti-NW-Zn samples. These results indicated that H_2_O_2_ can significantly increase the corrosion susceptibility of Ti alloys except for the Ti-NW-Zn materials, which made it a potential application for dental implants.

**Fig. 7:**
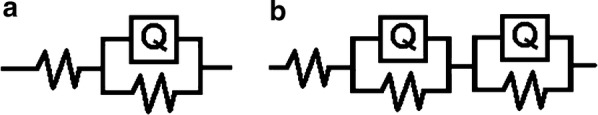
Model of equivalent circuit used for fitting the EIS results: **a** for cp-Ti and Ti-SLA; **b** for Ti-NW and Ti-NW-Zn

The spectra of cp-Ti and Ti-SLA samples immersed in solutions with different concentrations of H_2_O_2_ were interpreted with an equivalent circuit model of R_s_(R_p_Q) in Fig. 7a. As shown in Fig. 7b, the equivalent circuit model for Ti-NW and Ti-NW-Zn samples were R_s_(R_p_Q_p_)(R_t_Q_t_). Both of them are typical for the passive oxide layer. In these models, R_s_ represents the electrolyte resistance; R_p_ represents the surface oxide layer’s corrosion resistance, which is inversely proportional to corrosion rate; Q represents the constant phase elements (CPE) of the inter-barrier layer. The CPE, including Y_0_ and n, represents a shift from ideal capacitive behavior [35]. The corresponding R_p_, Y_0_-CPE and n values were listed in Tables 2 and 3. The corrosion tests showed that cp-Ti exhibited a statistically lower R_p_ value after immersion in solution with H_2_O_2_, especially in 30 mM H_2_O_2_. This result implied that the presence of H_2_O_2_ noticeably decreased the corrosion resistance of Ti alloys.

**Table 2:** Corrosion parameter values of cp-Ti exposed to different concentrations of H_2_O_2_

Substrates	Conditions	Impedance parameters (n = 3)			
		*R*_p_	Y_0_-CPE	*n*	χ^2^
cp-Ti	H_2_O_2_ = 0 mM	7.4E5 (2.0E5)	5.3	0.87	10^−3^
	H_2_O_2_ = 0.1 mM	9.1E5 (2.3E5)	4.5	0.87	10^−3^
	H_2_O_2_ = 0.5 mM	1.5E4 (3.4E3)	4.4	0.86	10^−3^
	H_2_O_2_ = 1.0 mM	1.3E4 (5.2E3)	4.1	0.90	10^−3^
	H_2_O_2_ = 5.0 mM	5.1E1 (1.4E1)	3.6	0.90	10^−3^
	H_2_O_2_ = 30.0 mM	1.8E−1 (9.0E−2)	3.8	0.93	10^−3^
	ANOVA	*p* < 0.01	–	–	–

Values: Mean (standard deviation); *R*_p_ (MΩ cm^−2^); Y_0_-CPE (μF cm^−2^)

**Table 3:** Corrosion parameter values of different substrates exposed to 30 mM H_2_O_2_

Substrates	Conditions	Impedance parameters (n = 3)						
		R_p_	Y_0_-CPE_Qp_	n	R_t_	Y_0_-CPE_Qt_	n	χ^2^
cp-Ti	H_2_O_2_ = 0 mM	7.4E5 (2.0E5)	5.3	0.87				10^−3^
cp-Ti	H_2_O_2_ = 30 mM	1.3E−1 (4.5E−2)	3.8	0.93				10^−3^
Ti-SLA	H_2_O_2_ = 30 mM	1.0E−1 (5.0E−3)	4.8	0.93				10^−3^
Ti-NW	H_2_O_2_ = 30 mM	3.5E−1 (5.8E−2)	4.9	0.92	34.6 (6.6)	4.4E−4	1	10^−3^
Ti-NW-Zn	H_2_O_2_ = 30 mM	3.1E0 (7.1E−1)	4.2	0.92	30.6 (3.4)	4.5E−4	1	10^−3^
	ANOVA	*p* < 0.01	–	–	–			

Values: Mean (standard deviation); *R*_p_ (MΩ cm^−2^); *R*_t_ (Ω cm^−2^); Y_0_-CPE_Qp_ (μF cm^−2^); Y_0_-CPE_Qt_ (μF cm^−2^)

Base metal alloys for dental applications are known to rely on their surface oxides for corrosion resistance in the oral environment. The surface oxide layer of Ti acts as a non-conductive barrier or resistor to electron flow between the metal and the electrolyte [36, 37]. In our present research, the XPS high-resolution spectra results confirmed that the oxide layer formed on the outermost surface of the cp-Ti was mainly composed of TiO_2_. Relative amounts of O and Ti on the cp-Ti surface were both reduced when exposure to H_2_O_2_. Therefore, it can be illustrated that the reduction of O and Ti levels in the oxide layer of cp-Ti resulted in a decrease in corrosion resistance (R_p_), and further increased the chemical reactivity and corrosion rates of the cp-Ti specimens.

Meanwhile, Table 3 revealed the corrosion tests of the four different surfaces exposed to H_2_O_2_. All samples presented a markedly drop of R_p_ value compared with cp-Ti in PBS group. In addition, Ti-SLA, Ti-NW and Ti-NW-Zn samples exhibited a statistically higher R_p_ value than cp-Ti surfaces after exposure to 30 mM H_2_O_2_, among which Ti-NW-Zn samples showed the highest value. These results demonstrated that H_2_O_2_ participated in breaking down the oxide layer over Ti alloys, while Ti-NW-Zn surfaces showed an excellent corrosion resistant to H_2_O_2_, which might be benefit from the zinc embed in their substrates.

Typical potentiodynamic polarization curves were observed for all surfaces in phosphate buffered saline (pH 7.4 at room temperature) with a small dose of H_2_O_2_ between 0 mM and 30 mM. The results of polarization tests showed large increases in the corrosion susceptibility of cp-Ti in a dose-dependent manner of H_2_O_2_. The polarization tests (V vs. Log I, Fig. 5d) showed that the surface of cp-Ti became much more susceptible to corrosion attack with the presence of H_2_O_2_, especially immersed in 30 mM H_2_O_2_. These results illustrated that the corrosion behavior of titanium surfaces was consistent with the Electrochemical Impedance Spectroscopies (EIS) results. Moreover, XPS analysis above mentioned supported our hypothesis that Ti alloys surfaces could be corroded when exposed to physiologically representative solutions with a small dose of H_2_O_2_.

We explored the typical potentiodynamic polarization curves for different surfaces in phosphate buffered saline (pH 7.4 at room temperature) with 30 mM H_2_O_2_. As shown in Fig. 6d. The corrosion potential of all four surfaces was significantly more electropositive under oxidizing conditions (H_2_O_2_) as compared to normal conditions (PBS), while no significant changes were observed on the Ti-NW-Zn surfaces. In addition, the corrosion current also significantly increased under oxidizing conditions (H_2_O_2_) for all surfaces, while the corrosion current of Ti-NW-Zn surfaces was the closest to the normal conditions (PBS alone). According to aforementioned results, it could be demonstrated that the H_2_O_2_ oxidizing environment did have different influences on the corrosion behavior of different titanium alloy surfaces. Among them, Ti-NW-Zn surfaces showed the lowest sensitivity to H_2_O_2_-induced corrosion attack, which might be attributed to the physiological role of Zinc element embed. Potential effects of Zinc element on Ti-NW-Zn material are needed to be illuminated in our further study.

### Cell biocompatibility

**Fig. 8:**
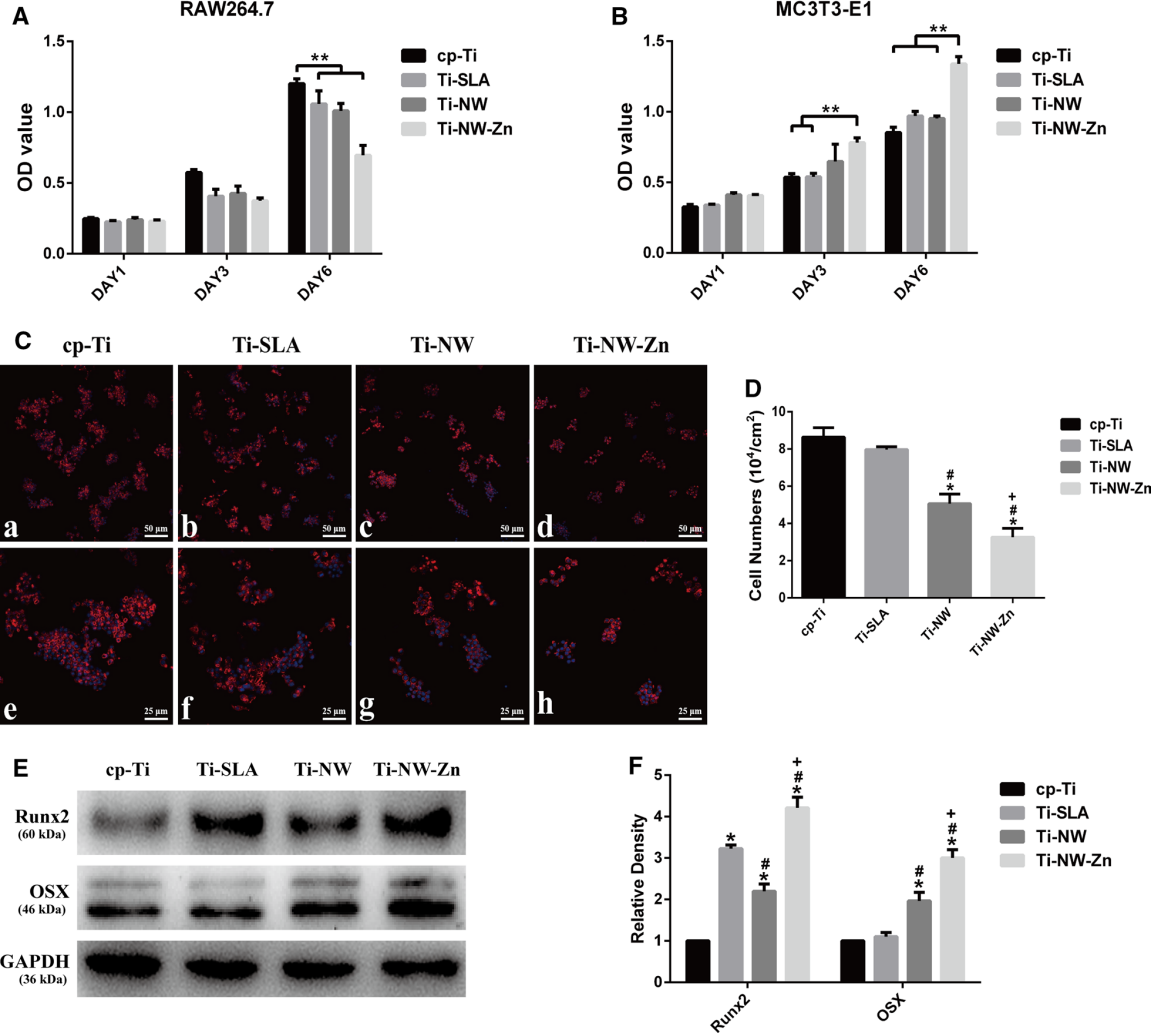
Cell biocompatibility and Osteogenic capacity test. **A**, **B** Proliferation of macrophage-like RAW264.7 cells and osteoblast-like MC3T3-E1 cells on four surfaces for 1, 3 and 6 days; **C** fluorochrome micrography of macrophages cultured on four surfaces. Actin (red), and cell nucleus (blue); **D** quantitative analysis for the number of macrophages on each surface; **E** expression levels of osteogenic markers Runx2 and OSX in osteoblast-like MC3T3-E1 cells on four surfaces (Runx2, Runt-related transcription factor 2; OSX, Osterix); **F** quantification of E by image J software. Results are presented as Mean ± SD, *p < 0.05 when compared with the cp-Ti group; ^#^*p* < 0.05 when compared with the Ti-SLA group; ^+^*p* < 0.05 when compared with the Ti-NW group

To examine the biocompatibilities of the four materials and their potential to the application of biomedical materials, macrophages and osteoblasts were seeded on the four different surfaces and cultured for 1, 3 and 6 days for proliferation tests. As shown in Fig. 8A, the proliferation of macrophage-like RAW264.7 cells were inhibited after 3 days culture when seeded onto the surfaces of Ti-SLA, Ti-NW and Ti-NW-Zn compared with the cp-Ti. Ti-NW-Zn surface showed an obviously negative effect on macrophage proliferation compared with other three surfaces after 6 days culture. Meanwhile, the proliferation of osteoblast-like MC3T3-E1 cells increased after 3 days culture when seeded onto Ti-NW-Zn surface compared with other three surfaces, while no significant differences were found between the cp-Ti, Ti-SLA and Ti-NW surfaces (Fig. 8B). These results showed that the four different surfaces had low toxicity to cells growth, and Ti-NW-Zn surface could even promote the proliferation of osteoblasts and inhibit cell growth of macrophages, exhibiting well biocompatibilities. Previous studies have shown that good biocompatibilities are the basis for cell growth and differentiation, and modification of material structures could influence different cell fates [38, 39]. In this study, it was proved that our modified surfaces had no toxicity to osteoblasts and were unsuitable for macrophage proliferation. Further investigation for macrophage morphology was carried out to explore the effects of different surfaces on cell adhesion. Macrophages were seeded on the four different surfaces after 1 day of culture. Differences in the cell morphology could be observed on the surfaces (Fig. 8C). Compared with those on the cp-Ti surfaces, cell spreading on Ti-NW and Ti-NW-Zn surfaces were less pronounced. Cells on Ti-SLA surfaces presented as a cluster, which was similar with that on cp-Ti surfaces. We also found that the number of adhered cells on the cp-Ti and Ti-SLA surfaces were significantly more than those on the Ti-NW and Ti-NW-Zn surfaces (*p* < 0.05) by counting the nuclei (Fig. 8D). However, no differences were observed between the cp-Ti and Ti-SLA surfaces and the cells attached to the Ti-NW-Zn surfaces were the least. All above results demonstrated that different surface morphology could have different cyto-compatibilities and influence macrophages behaviors. Ti-NW-Zn surface was proved to have good biocompatibility on osteoblasts development, while the macrophages growth was limited on this surface.

### Osteogenic capacity test

MC3T3-E1 osteoblast-like cell line was selected as the in vitro model of osteoblast development. The induction of the four material surfaces to the osteogenic differentiation of MC3T3-E1 cells was determined by Western blot (Fig. 8E, F). Many previous studies on osteoblast behaviors have shown that after 7-day culture, the ability of osteoblast differentiation and mineralization become obvious [40, 41]. In this study, protein expressions of Runx2 and OSX were significantly higher on Ti-SLA, Ti-NW and Ti-NW-Zn surfaces than those on cp-Ti surface after 7-day culture, indicating that Ti-SLA, Ti-NW and Ti-NW-Zn surfaces could all enhance the osteogenic differentiation, especially the Ti-NW-Zn surface, reflecting the good biocompatibilities.

Studies on the in vitro osteogenesis induction of bone implantation biomaterials are rarely reported. Wang et al. has found that titanium specimen with nanotubes (NTs) in small diameters (NT-30) could induce osteogenesis for the next-generation bone implant [42]. Zhao et al. reported that different topographies of Ti implants could influence the osteogenic capacity [43]. Recently, nanowire structures on titanium surfaces have been modified by many kinds of technologies. In this study, osteoblasts on Ti-NW-Zn surfaces was proved to have good osteogenic capacity and wonderful biocompatibility, suggesting the osseointegration potential of Ti implants.

### Macrophage polarization on different surfaces

The adhesion of macrophages has been proved above to produce highly oxidizing microenvironment between the cell membrane and metallic biomaterials surfaces by generating H_2_O_2_, which elevates the corrosion susceptibilities of material surfaces [17]. However, different surfaces might also have different effects on macrophages activation and polarization. It is well known that macrophage phenotypes have significant effects on biological performances of biomaterials [44]. Two macrophage phenotypes have been established in previous findings: the classical pro-inflammatory M1 and the alternative anti-inflammatory, wound healing M2. Thus, macrophage behaviors defined by the four different surfaces are needed to be evaluated. Previous studies have claimed that the surface nano-topography can reduce the infiltration of macrophages and attenuate the inflammatory process [45]. To uncover the immunomodulatory effects of different surfaces in our present study, macrophages polarization on the four surfaces were studied further.

**Fig. 9:**
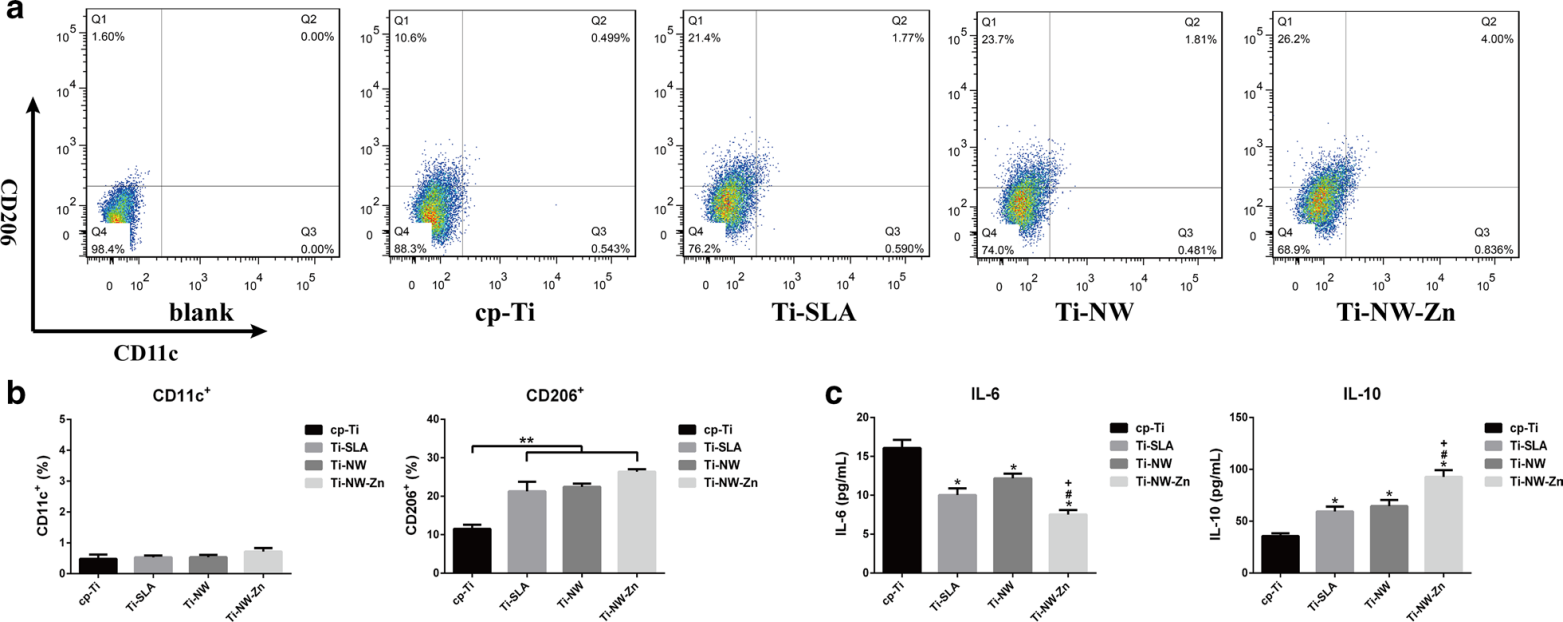
The effects of four surfaces on macrophage polarization in vitro. **a** The scatter plot of flow cytometry detection of M1 and M2 macrophage surface markers CD11c and CD206 on four surfaces; **b** the percentage of CD11c^+^ macrophages (M1) and CD206^+^ macrophages (M2) determined by flow cytometry on four surfaces; **c** cytokine release of M1 macrophage markers (IL-6) and M2 macrophage markers (IL-10) of macrophages on four surfaces. Results are presented as mean ± SD, *p < 0.05 when compared with the cp-Ti group; ^#^*p *< 0.05 when compared with the Ti-SLA group; (^+^*p* < 0.05 when compared with the Ti-NW group

From the results of flow cytometry (Fig. 9a), the distinct different polarization states were showed up in cp-Ti surface from other three surfaces. Specifically, the scatter plot of flow cytometry detection of CD11c (M1) and CD206 (M2), the surface markers of M1/M2 macrophages in Fig. 9b showed no difference in the percentage of CD11c^+^ macrophages (M1) on the four different surfaces. Nevertheless, the percentage of CD206^+^ macrophages (M2) on cp-Ti surface was significantly lower than that on Ti-SLA, Ti-NW and Ti-NW-Zn surfaces. However, there was no difference on the percentage of CD206^+^ macrophages (M2) between each other in Ti-SLA, Ti-NW and Ti-NW-Zn surfaces. These results suggested that the four different surfaces all exhibited the M2 phenotype, and Ti-SLA, Ti-NW and Ti-NW-Zn surfaces were more likely to induce the M2 states of macrophages than cp-Ti surface, especially the Ti-NW-Zn surfaces.

The main characteristics of polarized macrophages were the changed cytokine profiling. Enzyme-linked immunosorbent assays (ELISA) revealed the expression levels of inflammation-related cytokines in the four different surfaces at day 5 (Fig. 9c). Among them, IL-6 level had no difference between each surface, while IL-10 level in Ti-SLA, Ti-NW and Ti-NW-Zn groups were significantly higher than those in the cp-Ti group. IL-6 is recognized to be involved in pro-inflammation regulation, and IL-10 is the anti-inflammatory cytokine associated with the polarization of M2 macrophages and wound healing [46]. These results suggested that the preferences of macrophages in all four surfaces were tended to be the M2 phenotype rather than M1 phenotype, and Ti-SLA, Ti-NW and Ti-NW-Zn surfaces were more preferred to induce the M2 phenotype than the cp-Ti surface. It is indicated that titanium materials were of benefit to better healing and tissue reconstruction, which could be essential to the osseointegration.

According to aforementioned findings, it is concluded that different titanium surfaces could influence the status of surrounding macrophages activation. Specifically, all the four surfaces based on titanium were prone to induce M2 polarization of macrophages, contributing to the anti-inflammatory and pro-healing microenvironment. However, the four surfaces had no difference in the introduction of M1 polarization of macrophages that could lead to the pro-inflammatory and poor-healing microenvironment. It is known that the M2 polarization of macrophages is beneficial for wound healing, tissue remodeling, and osteogenesis, while the M1 macrophages indicate consistent inflammatory reactions [47]. Based on the above results, the titanium surfaces, especially the Ti-NW-Zn surfaces in this study seemed favorable to bone formation. Zinc element is believed to exert a vital role in this process.

### Antioxidant enzyme relative genes expression

**Fig. 10:**
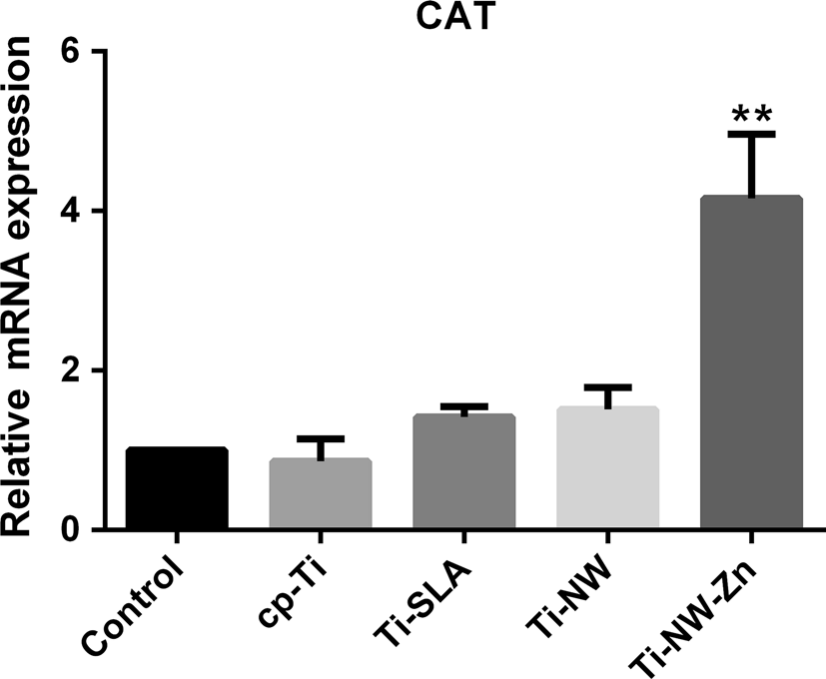
Antioxidant enzyme relative gene expression on cp-Ti, Ti-SLA, Ti-NW and Ti-NW-Zn. There were significant differences in the gene expression of CAT. Results were presented as mean ± SD (*p < 0.05, **p < 0.01)

To further investigate the mechanism of corrosion behavior of Ti-NW-Zn surface, the antioxidant systems in macrophages on different surfaces were detected. Catalase (CAT), an enzyme found mainly in peroxisomes, can degrade hydrogen peroxide to water and oxygen and thus exert vital roles in resistance to oxidative cell injury [48]. Meanwhile, CAT inhibits the release of hydrogen peroxide into extracellular matrix and reduce oxidative stress reaction on the metal surfaces [49]. Here, Ti-NW-Zn samples obviously increased CAT expression in attached macrophages with more than fourfolds change compared to the other three surfaces (Fig. 10). These results reflected that Ti-NW-Zn samples might reduce the accumulation of hydrogen peroxide by regulating the expressions of antioxidant enzymes such as CAT in macrophages, which might be attributed to the Zinc element embed into the its base.

Zinc is reported to have an intensive effect on the organization of cellular glutathione, which is vital to cellular antioxidant defense [31]. Also, zinc could attenuate the activities of oxidant promoting enzymes such as iNOS, and stimulate the pro-inflammatory M1 macrophage phenotype, suggesting that zinc element inhibits the M1 phenotype of macrophages and the inflammation. These previous reports supported our results that Ti-NW-Zn surface was more preferred to induce the M2 phenotype for better healing and tissue reconstruction. Meanwhile, zinc could suppress the generation of ROS products, while increase the activation of antioxidant enzymes such as CAT and SOD [50], and zinc could induce the synthesis of metallothionein, a powerful scavenger of free radicals [51]. Thus, zinc not only defends cells against oxidative injuries and inflammation, but also protects metal surfaces from macrophage-induced oxidation corrosion such as hydrogen peroxide.

The detection of antioxidant enzyme system in our research explained the relationship between antioxidant enzyme and hydrogen peroxide induced by macrophages, which were consistent with the corrosion behavior results showed above. Furthermore, our results also revealed that Ti-NW-Zn sample might be one of the potential materials for antioxidant properties.

## Conclusions

We compared the corrosion behaviors of four common titanium materials (cp-Ti, Ti-SLA, Ti-NW and Ti-NW-Zn) with excellent biocompatibilities and osteogenic capacities under the peroxygen environment created by macrophages. It was found that titanium surfaces exposure to a high oxidizing environment would suffer the risk of corrosion, which could be aggravated by the macrophages around titanium implants. Furthermore, we found that zinc did serve to ameliorate hydrogen peroxide induced oxidation corrosion on Ti surfaces by up-regulating CAT level and reducing generation of hydrogen peroxide. Besides, zinc embedded Ti surfaces changed the adhesion ability and polarization of macrophages to further induce anti-inflammatory and wound healing M2 phenotype on metal surfaces. Thus, compared with other three surfaces, Ti-NW-Zn surface was proved to be benefit to better bone formation, maintain the status of the excellent antioxidant defense system, and reduce corrosion susceptibility of the titanium material in the oxidizing microenvironment.

## Abbreviations

Ti: titanium
cp-Ti: commercially pure titanium
Ti-SLA: sandblasting and acid etching-modified titanium
Ti-NW: nanowires-modified titanium
Ti-NW-Zn: zinc-containing nanowires-modified titanium
ROS: reactive oxygen species
H_2_O_2_: hydrogen peroxide
CAT: catalase
